# *Pneumocystis jirovecii* Pneumonia in Children with Hematological Malignancies: Diagnosis and Approaches to Management

**DOI:** 10.3390/jof6040331

**Published:** 2020-12-02

**Authors:** Elpis Mantadakis

**Affiliations:** Department of Pediatrics, Hematology/Oncology Unit, University General Hospital of Alexandroupolis, Democritus University of Thrace, 68 100 Alexandroupolis, Thrace, Greece; emantada@med.duth.gr; Tel.: +30-25513-51411; Fax: +30-25510-30340

**Keywords:** *Pneumocystis jirovecii*, *Pneumocystis jirovecii* pneumonia (PJP), children, leukemia, cotrimoxazole, dapsone, pentamidine, atovaquone, bronchoalveolar lavage fluid (BAL)

## Abstract

*Pneumocystis jirovecii* pneumonia (PJP) is an opportunistic infection that mostly affects children with suppressed cellular immunity. PJP was the most common cause of infectious death in children with acute lymphoblastic leukemia prior to the inclusion of cotrimoxazole prophylaxis as part of the standard medical care in the late 1980s. Children with acute leukemia, lymphomas, and those undergoing hematopoietic stem cell transplantation, especially allogeneic transplantation, are also at high risk of PJP. Persistent lymphopenia, graft versus host disease, poor immune reconstitution, and lengthy use of corticosteroids are significant risk factors for PJP. Active infection may be due to reactivation of latent infection or recent acquisition from environmental exposure. Intense hypoxemia and impaired diffusing capacity of the lungs are hallmarks of PJP, while computerized tomography of the lungs is the diagnostic technique of choice. Immunofluorescence testing with monoclonal antibodies followed by fluorescent microscopy and polymerase chain reaction testing of respiratory specimens have emerged as the best diagnostic methods. Measurement of (1-3)-β-D-glucan in the serum has a high negative predictive value in ruling out PJP. Oral cotrimoxazole is effective for prophylaxis, but in intolerant patients, intravenous and aerosolized pentamidine, dapsone, and atovaquone are effective alternatives. Ιntravenous cotrimoxazole is the treatment of choice, but PJP has a high mortality even with appropriate therapy.

## 1. Introduction

*Pneumocystis jirovecii* is an important cause of pneumonitis in immunocompromised children. The organism was initially described by Chagas and Delanoes but was later named in honor of Antonio Carini, who isolated it from infected rats [1]. Since the genus incorporates various species that are host-specific, the organism causing human pneumonia and previously known as *P. carinii* was renamed as *P. jirovecii*, in honor of Otto Jirovec, a Czech Professor of Parasitology, who first isolated the organism from humans [2,3].

*Pneumocystis* was initially misclassified as a trypanosome and later as a protozoan because of the morphologic features of the small trophic form, the larger cyst form, and the rupture of its cyst to release new trophozoites. However, an analysis of the small ribosomal RNA subunit clearly established a phylogenetic linkage to the fungal kingdom, and all subsequent studies have verified that *Pneumocystis* is a fungus [4]. 

In 2014, the first successful cultivation and propagation of *Pneumocystis* directly from bronchoalveolar lavage (BAL) fluid was achieved, using a three-dimensional air–liquid interface culture system formed by a respiratory epithelial cell line [5]. While this represents a breakthrough and provides the potential to perform antifungal susceptibility testing [6], the process still requires cell culture and confirmation of the method by other laboratories, severely limiting its use in everyday diagnostics.

## 2. *P. jirovecii* in Human Disease

Pneumocystis first came to attention as a cause of pneumonitis in malnourished infants in the 1940s [7]. Before the 1980s, a few dozen cases of *P. jirovecii* pneumonia (PJP) were reported annually in USA, almost exclusively in patients with cancer receiving chemotherapy without chemoprophylaxis [8]. Since 1981, PJP has been recognized as an acquired immunodeficiency syndrome (AIDS)-defining illness and the most common opportunistic infection in Human Immunodeficiency Virus (HIV)-positive patients [9].

PJP is almost never observed in immunocompetent persons. In recent years, the diagnosis of PJP has increased among non-HIV patients [10,11], particularly among those who receive immunosuppressive therapy for hematological malignancies [12,13], solid tumors, collagen vascular diseases [14], and hematopoietic or solid organ transplantation [15]. In a single-center study from Denmark of 604 patients with PJP, the ratio of non-HIV versus HIV patients increased from 1.7 to 5.6 during the 9 year study period [16]. A British study also demonstrated that the laboratory-confirmed cases, deaths, and hospital admissions from PJP in England increased by an average of 7% per year from 2000 to 2010, but this surge was limited to patients with hematological malignancies or who had been transplanted [17]. 

In children with leukemia, PJP was by far the most common cause of infectious death prior to the inclusion of PJP prophylaxis as part of the standard medical care [18]. PJP is one of the 14 acute toxic effects of antileukemic therapy recognized by Delphi consensus by 15 international childhood acute lymphoblastic leukemia (ALL) study groups [19]. Children with brain neoplasms receiving high-dose corticosteroids or methotrexate are also at increased risk for PJP [20,21]. In fact, nowadays, chronic corticosteroid use is the single most important risk factor for patients without AIDS who develop PJP. A daily dose of corticosteroids 16 mg of prednisone equivalent for a period 8 weeks is associated with a significant risk of PJP [22]. In assessing a patient’s overall risk for PJP, the clinician should weigh not only the use of corticosteroids, but also consider the underlying disease itself, and its therapy. For example, methotrexate, an essential antileukemic drug in ALL, is independently associated with increased risk of developing PJP in patients with rheumatoid arthritis [23].

Besides children with AIDS and cancer, PJP is rare, even among children receiving long-term corticosteroid therapy. In a retrospective cohort of patients ≤18 years of age who received at least two prescriptions for a systemic glucocorticoid within a 60-day period and did not have cancer, AIDS or, history of transplantation, the incidence of PJP was 0.61 and 0.53 per 10,000 patient-years in those exposed and unexposed to PJP prophylaxis, respectively [24].

The epidemiology of PJP in children with hematologic malignancies and other types of cancer has not been thoroughly studied. A prospective survey of the regimens adopted for PJP prophylaxis in Italian centers of pediatric hematology–oncology showed the cumulative incidence of PJP at 3 years to be only 0.09% [25]. A retrospective cohort study of children with newly diagnosed cancer between January 2004 and December 2009 at 45 U.S. children’s hospitals disclosed 33,067 cancer patients, 169 (0.5%) of whom developed PJP. Patients with ALL and acute myeloid leukemia (AML) had the highest relative risks of PJP, when compared with other types of cancer [26].

Children with high-risk ALL or high-grade non-Hodgkin lymphomas that receive intensely immunosuppressive regimens are at an exceptionally high risk for PJP [27]. Children with Hodgkin’s lymphoma are also at risk because they are frequently lymphopenic at diagnosis or will become lymphopenic with chemotherapy [28]. Children undergoing hematopoietic stem cell transplantation (HSCT) are also at high risk for developing PJP. The Center for International Blood and Marrow Transplant Research evaluated the incidence, timing, prophylactic agents, risk factors, and mortality of PJP after autologous and allogeneic HSCT. Overall, 0.63% of allogeneic and 0.28% of autologous recipients of their first HSCT developed PJP [29]. Risk factors for PJP infection included lymphopenia and mismatched HSCT. Overall, survival was significantly poorer among cases versus controls; risk factors included graft versus host disease (GVHD) and poor immune reconstitution [29].

## 3. Clinical Picture and Imaging Findings of PJP

*P. jirovecii* is transmitted through the airborne route, and most children worldwide have been exposed to the organism before their fourth birthday [30,31]. In children with leukemia, PJP has a high mortality even with prompt therapy [18,32]. Extrapulmonary involvement is rare compared with in patients with advanced AIDS. In the lungs, the trophic form of *P. jirovecii* attaches to type I alveolar epithelial cells, which allows the fungus to be transformed into its cystic form [33]. Asymptomatic lung colonization is common in patients with normal immunity. Human-to-human spread is well documented [34,35,36], and both asymptomatic colonizers and patients with pneumonia can transmit the fungus to immunocompromised people. Molecular epidemiologic studies have suggested that active infection may be due to reactivation of latent infection or due to recent acquisition from environmental exposure [35,37,38].

PJP is characterized by the presence of intra-alveolar foamy exudate, diffuse alveolar damage, and interstitial lymphoid infiltrates [39]. It is the host’s inflammatory response that causes significant lung injury [40], and this response is more pronounced in children with leukemia than in children with AIDS. All signs and symptoms of PJP are nonspecific, and a high index of suspicion is required for early diagnosis. Leukemic children with PJP at presentation may have fever with or without chills, nonproductive cough, tachypnea, intercostal retractions, nasal flaring, weight loss, and progressive exertional dyspnea [41]. Hemoptysis can occur but is rare, while cyanosis occurs in cases with severe hypoxemia. Physical examination reveals mild crackles and rhonchi, but the results can be surprisingly normal in many patients despite profound dyspnea. Intense hypoxemia—out of proportion to that expected based on the clinical picture—with an increased alveolar–arterial oxygen gradient, respiratory alkalosis, and impaired diffusing capacity of the lungs are hallmarks of PJP. In general, patients with leukemia and PJP have more acute and severe disease compared with patients with AIDS [42,43]. However, as the knowledge of PJP in HIV-uninfected patients has increased and the laboratory diagnosis perfected, it is now more common to see pneumonia in leukemic patients presenting with more indolent course [44].

On chest radiography, diffuse bilateral lung infiltrates extending from the perihilar region are seen in most patients, but unilateral or asymmetric infiltrates can occur. Compared to patients with bacterial pneumonia, patients with PJP are more likely to have bilateral lung disease, higher frequency of hypoxemia, and lower frequency of pleural involvement [41]. However, no radiological findings are pathognomonic of PJP.

Depending on the presenting symptoms and oxygen saturation by pulse oximetry, PJP can be stratified into mild, moderate, and severe [32]. In severe PJP, dyspnea and tachypnea are present at rest, oxygen saturation is 91%, and chest radiography usually demonstrates extensive bilateral interstitial lung changes along with diffuse alveolar shadowing. The need for mechanical ventilation in patients with severe PJP dramatically increases the risk of death [45].

High-resolution computerized tomography (HRCT) is the best radiological method for diagnosis of PJP and should be considered, even if chest radiographs are normal [46]. On HRCT, PJP typically presents with bilateral mosaic regions of ground-glass attenuation and thickening of the interlobar septa [46,47]. Bilateral ground-glass opacities on the upper lobes with peripheral sparing are the typical imaging findings in non-HIV immunocompromised patients [48]. Lymphadenopathy is uncommon. In immunocompromised patients, bronchial wall thickening is more indicative of bacterial pneumonia, presence of nodules is more characteristic of fungal infection, and a mosaic pattern is more indicative of PJP [49]. Different immune reactions to *P. jirovecii* in patients with and without AIDS lead to different radiographic patterns. Thus, air cysts, pneumothorax, and pneumomediastinum are much less common in patients with leukemia compared with patients with AIDS [50]. In adults, the mean lung attenuation (MLA) on CT scans highly correlates with Acute Physiology and Chronic Health Evaluation II scores and the need for mechanical ventilation, but MLA has limited value in predicting patient mortality [51]. For diagnostic purposes, decreased diffusion capacity of carbon monoxide (DLCO) is useful, since PJP is highly unlikely if DLCO is normal [52]. When the CT findings are unclear, fluorodeoxyglucose positron emission tomography (FDG PET) can be used to diagnose PJP, and it also aids with monitoring response to therapy [53,54,55].

Differences in the laboratory features, clinical presentation, and imagining findings of children with PJP and leukemia versus AIDS are summarized in Table 1.

## 4. Laboratory Diagnosis of PJP

Serum lactate dehydrogenase (LDH) is typically elevated in patients with AIDS and PJP, but a single-center retrospective analysis identified initial LDH values as a tool to identify patients at high risk of death irrespective of HIV status [57]. As mentioned previously, culturing *P. jirovecii* has proven very difficult and is not a routine diagnostic investigation. This creates a major problem with PJP diagnosis since there is a lack of a true gold standard diagnostic assay. In the past, the best assay for confirming a diagnosis of PJP was the histological identification of its trophic forms and cysts in clinical specimens using a variety of different stains. Giemsa, Diff-Quik (a modified Giemsa stain with very short preparation time), crystal violet, Wright, Gomori methenamine silver (GMS), and Toludine blue O (TBO) have traditionally been used, but have decreased sensitivity compared with immunofluorescence antibody testing (IFAT) [58,59]. Giemsa, Diff-Quik, crystal violet, and Wright stains can detect both the trophozoite and cyst forms of the fungus, while GMS and TBO selectively stain the wall of *Pneumocystis* cysts [60]. IFAT with labelled monoclonal antibodies followed by fluorescent microscopy has surpassed conventional microscopy due to its superior sensitivity [61], especially when testing BAL fluid as compared with samples from the upper airways. If IFAT is unavailable, Giemsa or TBO should be used as the primary staining method, with confirmation of the results with GMS [62]. All staining methods are limited by the quality of the respiratory specimens and the experience of the laboratory personnel. The isolated use of microscopy for diagnosis should be avoided due to its low sensitivity and specificity and its inability to differentiate colonization from true pneumonia. Interpretation of IFAT assays may be difficult due to artifacts that can easily be confused with trophic forms or cysts by inexperienced laboratory staff [32]. Moreover, microscopy and cyst-staining assays can fail to detect PJP infection with mostly trophic forms of the fungus. Finally, microscopy is less sensitive to detect *P. jirovecii* in children with leukemia, who typically carry lower fungal loads than patients with AIDS [63].

Over the last decades, polymerase chain reaction (PCR) has been developed for the diagnosis of PJP in HIV and non-HIV patients, and PCR is currently included in the guidelines for the diagnosis of PJP in febrile neutropenic patients with hematological malignancies [64]. It can be used in fresh or formalin-fixed, paraffin-embedded samples [65]. It has superior sensitivity compared with immunofluorescent microscopy, but its specificity varies. By combining multiple tests, PJP can be more confidently excluded if all tests are negative and confirmed if all tests are positive in children with leukemia and HSCT recipients [66]. Positive PCR results need careful interpretation if microscopy is negative, while negative PCR results in the face of positive microscopy are technically inconsistent and likely represent artifacts or false-negative results due to the presence of PCR inhibitors in respiratory samples [66,67].

The use of conventional and nested PCR techniques has for the most part been replaced by real-time PCR, which allows quantification in clinical samples and improved specificity [68]. PCR has close to 100% negative predictive value, and a negative result can reliably exclude PJP [69,70]. Efforts are ongoing to improve the specificity of this molecular method in differentiating PJP from colonization. While successful attempts have been made to define a threshold to distinguish between pneumonia and colonization [71], these are hindered by the various molecular targets, type of sample, and volume of BAL obtained. Many gene targets have been used, but the mitochondrial large subunit of ribosomal RNA has been the most commonly used target [72]. Other targets include the short subunit of ribosomal RNA, the major surface glycoprotein [73,74], and the enzymes dihydrofolate reductase and dihydropteroate synthetase, which, however, are more commonly used for genotyping in epidemiological studies or in patients who fail cotrimoxazole therapy [75]. In summary, quantitative PCR can reliably exclude PJP, but a positive result should be interpreted in the context of the clinical signs and the patient’s immune suppression.

(1-3)-β-D-glucan (BDG) is a cell wall polysaccharide present in most fungi, including *Candida* spp., *Aspergillus* spp., and *P. jirovecii* (but not the Zygomycetes) that can be detected in serum/plasma using various commercially available assays [76,77]. Although it lacks specificity, patients negative by BDG or both BDG and PCR are extremely unlikely to have PJP [78]. BDG testing of BAL fluid does not add to the testing of serum and is not recommended [79]. In 31 HIV-negative, immunocompromised patients suspected of PJP on the basis of clinical presentation and chest imaging, elevated serum BDG was a reliable indicator of PJP with a sensitivity of 90% and a specificity of 89% at the 60 pg/mL cut-off [80]. In PJP- positive patients, BDG serum levels decrease during effective treatment. Preferably, BDG positivity should be combined with one of the *P. jirovecii*-specific assays described above, because many other fungal pathogens in children with hematological malignancies can provide high BDG serum concentrations. Surfactant protein-D is another biomarker of PJP that continues to increase after failure of treatment and may be associated with clinical outcome in non-HIV patients with PJP [81].

Open lung biopsy is the most sensitive and specific diagnostic procedure [82] but is rarely performed nowadays, especially in children with leukemia that are frequently severely ill, thrombocytopenic, and/or suffer from coagulopathy, when they present with a new-onset pulmonary infiltrate. 

## 5. Differential Diagnosis of PJP

The differential diagnosis of PJP in a child with leukemia is extensive and includes cytomegalovirus (CMV) and other viral pneumonias, lymphocytic interstitial pneumonia, atypical bacterial pneumonia due to *Mycoplasma pneumoniae* and *Chlamydia pneumoniae*, legionellosis, lung infection by *M. tuberculosis* or by nontuberculous mycobacteria, pulmonary embolism, and pulmonary GVHD. PJP can occur in conjunction with CMV in non-HIV immunocompromised patients with respiratory failure, and mortality may be increased in the setting of coinfection [83]. Coinfection with CMV was significantly more common in adult patients who were receiving corticosteroids and T-cell immunosuppressants, compared with corticosteroids alone [84].

## 6. Prophylaxis against PJP

The Centers for Disease Control and Prevention recommends patients with PJP not to have direct contact with other immunosuppressed patients [85]. Protective isolation decreases but does not eliminate the risk of PJP, as shown by a retrospective survey in which 12 of 55 adults with hematological malignancies who developed PJP were on protective isolation in a laminar airflow room [86].

Without chemoprophylaxis, up to 25% of pediatric oncology patients receiving chemotherapy will develop PJP [18]. Pioneering studies in the 1970s demonstrated the central role of cotrimoxazole in the prevention of this serious infection [87]. Cotrimoxazole prophylaxis for PJP is so successful that nowadays noncompliance with drug-taking is the main reason for pneumonia. This is more common in older children and adolescents with cancer and poor obedience with the administered therapy [88]. Hughes et al., in a double-blinded, placebo-controlled clinical trial conducted at St. Jude Children’s Research Hospital (SJCRH), randomized 160 children and young adults with cancer to receive placebo or 150 mg of trimethoprim and 750 mg of sulfamethoxazole per square meter of body surface area per day. Remarkably enough, 17 of 80 patients receiving placebo developed PJP versus none of the 80 patients given cotrimoxazole [89]. The same investigators conducted a two-year randomized clinical trial in children and adolescents with ALL to assess the efficacy of cotrimoxazole for PJP prophylaxis when given on three consecutive days per week versus daily. PJP did not develop in any of the 92 patients receiving the drug daily or in any of the 74 patients who received it on three consecutive days per week [90]. These results were confirmed by a study of the University of Milan, Italy [91]. Since cotrimoxazole intolerance with the above regimen, especially hematological toxicity, can occur and may interfere with the administration of methotrexate and 6-mercaptopurine in children receiving maintenance chemotherapy for ALL, lower doses of cotrimoxazole, that is, 18 mg/kg/day, daily or on three consecutive days per week have been successfully used for PJP prophylaxis in the Netherlands [92].

PJP prophylaxis should be considered for all allogeneic HSCT recipients and for autologous recipients who have leukemia or lymphoma, are receiving intense conditioning, or have recently received fludarabine or 2-chlorodeoxyadenosine [93]. Balanced against serious adverse events of cotrimoxazole, PJP prophylaxis in children is indicated at very low PJP incidence rates, while in adults when the risk for pneumonia exceeds >3.5% [94].

According to the European Conference on Infections in Leukemia (ECIL) guidelines, standard indications for PJP prophylaxis are as follows: ALL patients from induction to end of maintenance chemotherapy, allogeneic HSCT recipients from engraftment to 6 months and as long as receiving immunosuppressive therapy, recipients of alemtuzumab, and recipients of corticosteroids >0.4 mg/kg/day or 16 mg/day of prednisone equivalent for >1 month. Optional indications are lymphoma patients treated with R-CHOP14 or escalated BEACOPP, patients on fludarabine, cladribine, or mycophenolate mofetil, patients with primary brain tumors or brain metastases on high-dose corticosteroids, and patients with AML and solid tumors throughout the duration of chemotherapy [95].

Intermittent dosing of cotrimoxazole on two instead of three consecutive or nonconsecutive days per week is also effective in children with leukemia and lymphoma [96,97,98,99]. A prospective survey of the regimens adopted for PJP prophylaxis in all children with cancer treated at Italian centers of Pediatric Oncology showed that even a single-day per week course of prophylaxis with cotrimoxazole at 5–10 mg/kg/week for trimethoprim may be sufficient to prevent PJP in children with cancer undergoing intensive chemotherapy [25].

In spite of the above, occasionally patients with hematological malignancies still fail prophylaxis and develop pneumonia [56]. A systematic review of children with ALL and adults with acute leukemia, solid organ transplantation, and autologous bone marrow transplantation estimated that the number of patients needed to treat to prevent PJP is 19 (95% confidence intervals 17 to 42) [100].

Intravenous or aerosolized pentamidine, oral dapsone, and atovaquone are acceptable alternatives for children unable to tolerate cotrimoxazole due to an allergic reaction or hematological toxicity. Table 2 summarizes studies of children with cancer who underwent HSCT and received intravenous pentamidine for PJP prophylaxis [101,102,103,104,105,106,107,108,109]. 

Similar results for adults undergoing HSCT have been published illustrating efficacy, safety, and patient approval of intravenous pentamidine for PJP prophylaxis [110,111], although some have questioned the efficacy of intravenous pentamidine in children undergoing chemotherapy [112].

Regarding aerosolized pentamidine for PJP prophylaxis, beyond the SJCRH study [101], limited data are available. Aerosolized pentamidine was administered to 22 children with acute leukemia during maintenance chemotherapy or post-Bone Marrow Transplantation (BMT) and was adequately tolerated and efficacious, since none of the patients developed PJP during the 3.5 years of close observation [113]. Investigators at the University of Texas Southwestern Medical Center at Dallas administered monthly aerosolized pentamidine 200 mg/m^2^ to 60 children with malignancies who had experienced severe adverse reactions to cotrimoxazole; they found that none of the treated patients developed PJP during more than a year of observation. However, adverse reactions including bronchospasm, cough, vomiting, and nausea occurred during 10% of the treatments, although only three patients (5%) discontinued therapy [114]. In adults, two large studies show that aerosolized pentamidine is clearly inferior to cotrimoxazole in preventing PJP in the post-transplantation setting for hematologic malignancies, aplastic anemia, or myelodysplasia and is associated with a higher mortality at one year after transplantation [115,116]. Besides, aerosolized pentamidine is frequently avoided in children <5 years of age due to concerns regarding their ability to inhale the entire dose.

Considering dapsone, its weekly oral administration has been feasible and effective for prevention of PJP because of its prolonged plasma half-life and the long incubation period and replication cycle of *P. jirovecii* [117]. Maltezou et al. reviewed 33 children who underwent BMT in whom dapsone 50 mg/m^2^ once a week was used for PJP prophylaxis due to cotrimoxazole intolerance. After a median follow-up of one-year post-BMT, no proven cases of PJP were diagnosed [118]. Sangiolo et al. compared 155 HSCT recipients on dapsone prophylaxis with 310 matched control patients who received cotrimoxazole throughout the post-transplantation course. Two of 155 patients developed PJP versus 0 of 310 controls [119]. Souza et al. evaluated the effectiveness of dapsone prophylaxis 50 mg orally twice daily for three days per week to twice weekly cotrimoxazole in preventing PJP after allogeneic HSCT in 646 adults who received allogeneic transplantation. The incidence of PJP was 0.37% for cotrimoxazole versus 7.2% for dapsone, a highly significant difference that was not accounted for by age, donor source, chronic GVHD, or disease relapse [120]. Hence, extreme caution is required in the transplantation setting when using an alternative to cotrimoxazole regimens for PJP prophylaxis. If dapsone is used, the patient should be tested for glucose-6-phosphate dehydrogenase (G6PD) deficiency because the drug can cause hemolysis [121]. Moreover, heterozygosity for cytochrome b5 reductase deficiency may predispose children with leukemia to methemoglobinemia even on a thrice-weekly regimen of dapsone [122,123]. Finally, the drug is metabolized via the hepatic P-450 system; thus, it can interfere with cyclosporine metabolism in transplanted patients and in children with leukemia who receive prophylactic or therapeutic antifungal azoles.

Another alternative to cotrimoxazole for PJP prophylaxis in children with leukemia is atovaquone. A SJCRH study reviewed 86 children with ALL or AML who received atovaquone daily for PJP prophylaxis due to intolerance to cotrimoxazole. PJP was not diagnosed in any patient [124]. Atovaquone is very expensive and requires ingestion with fatty foods for optimal absorption. Common side effects include headache, skin rash, and gastrointestinal disturbances, but bone marrow toxicity is uncommon. Prophylactic regimens for PJP in children with leukemia and HSCT recipients are summarized in Table 3 [95,125].

Notably, guidelines have been published that recommend all children with malignancies undergoing chemotherapy, including those with solid tumors, receive prophylaxis with cotrimoxazole, unless well-defined contraindications exist [126].

## 7. Treatment of PJP

Early diagnosis and treatment of PJP are crucial for survival [127]. *P. jirovecii* is extremely resistant to traditional antifungal agents, including polyenes and azoles [128]. On the other hand, animal and limited clinical data indicate that echinocandins have activity against *P. jirovecii* [129,130]. The European Organization for Research and Treatment of Cancer, the International Immunocompromised Host Society, and The European LeukemiaNet have jointly published guidelines for the treatment of PJP in non-HIV-infected hematology patients [131]. Treatment of choice for PJP remains intravenous cotrimoxazole, that is, trimethoprim 15–20 mg/kg/day and sulfamethoxazole 75–100 mg/kg/day divided in two to four daily doses. Treatment should be initiated immediately after obtaining appropriate samples, that is, nasopharyngeal aspirates in infants [132], sputum in adolescents, and BAL with fiberoptic bronchoscopy in children of all ages [66]. Fiberoptic bronchoscopy is generally a safe and highly diagnostic procedure in the immunocompromised host with pneumonia due to opportunistic pathogens [133].

Documenting the serum levels of trimethoprim or sulfamethoxazole is useful in critically ill patients receiving intravenous cotrimoxazole therapy. In individual patients with persistent PJP despite adequate therapy with cotrimoxazole, search for mutations in the genes for dihydropteroate synthase or dihydrofolate reductase may be justified [134]. However, *P. jirovecii* genotypes are unlikely to have clinical relevance in most cases [135,136].

Treatment with intravenous cotrimoxazole is continued for three weeks, and clinical improvement is usually apparent after seven days of therapy. If clinical improvement does not develop within a week, a second infection or resistance to cotrimoxazole should be considered, and the diagnostic procedures repeated. In case of allergy to sulfa drugs or clinical deterioration on cotrimoxazole, second-line agents should be used, including primaquine plus clindamycin [137], atovaquone, or intravenous pentamidine. Primaquine should not be used in children with G6PD deficiency due to the risk of hemolysis [138], while clindamycin can cause pseudomembranous colitis [139]. Aerosolized pentamidine should only be reserved for children with mild disease who cannot tolerate other therapies. Therapeutic regimens for PJP in children with leukemia and HSCT recipients are summarized in Table 4.

Patients who have been successfully treated for PJP should receive secondary prophylaxis to prevent recurrence. Drugs of choice for secondary PJP prophylaxis are intermittent oral cotrimoxazole or monthly aerosolized pentamidine.

Adjunctive corticosteroids in addition to cotrimoxazole are of substantial benefit to HIV-infected patients with moderate-to-severe PJP defined by the degree of hypoxemia. In one retrospective study of non-HIV adults with severe PJP, a dose of prednisone 60 mg/day was also associated with better outcome [140]. However, other observational studies do not support the use of corticosteroids in HIV-uninfected immunocompromised patients [141]. Hence, given the lack of randomized controlled clinical trials, the decision regarding the use of corticosteroids in children with hematological malignancies and PJP should be individualized. The American Thoracic Society advises adding corticosteroids into the treatment of HIV-uninfected patients with moderate-to-severe PJP [142], but the German Society of Hematology and Medical Oncology does not recommend corticosteroids in this setting [64]. Finally, extracorporeal membrane oxygenation [143] and adjunctive surfactant treatment [144] have been successfully used in carefully selected children with PJP.

## 8. Conclusions

PJP is a life-threatening opportunistic infection in children with hematological malignancies receiving chemotherapy [101]. In high-risk children with leukemia, the clinical and radiological findings are usually enough for initiating empirical therapy, but every effort should be made to achieve a mycological diagnosis. IFAT and PCR of respiratory samples are now the reference diagnostic methods, but serum BDG testing is invaluable to rule out *P. jirovecii* as a cause of pneumonia. Oral cotrimoxazole two or three days per week is highly effective for prevention of PJP. However, given its potential for allergic reactions and hematological toxicity, other alternative drugs should be carefully considered for PJP prophylaxis in selected children with leukemia. Antimicrobial resistance of *P. jirovecii* appears to be clinically rare, and although in vitro cultivation of the organism for susceptibility testing has been feasible since 2014, it remains unavailable for everyday clinical diagnostics. Finally, the population of children at risk for PJP will likely increase in the future beyond children with hematological malignancies to include pediatric patients with a variety of autoimmune and inflammatory disorders treated with corticosteroids, anti-Tumor Necrosis Factor agents, and calcineurin inhibitors [145,146,147].

## Figures and Tables

**Table 1:** Differences in laboratory features, clinical presentation, and imagining findings of children with *Pneumocystis jirovecii* pneumonia (PJP) and underlying leukemia versus AIDS.

Feature	PJP in Children with Leukemia	PJP in Children with AIDS
Number of organisms in BAL fluid	Low	High
Clinical presentation	Acute, severe	Subacute
Air cysts, spontaneous Pneumothorax and pneumomediastinum	Uncommon	Common
Possible diagnostic delay	Usually longer	Shorter
Mortality *	28–53%	17–30%

* From Table 2 of reference [56]. BAL: bronchoalveolar lavage.

**Table 2:** Series of pediatric patients with malignancies or who underwent hematopoietic stem cell transplantation (HSCT) and received pentamidine intravenously for PJP prophylaxis.

		Ref.	Country	Study Period	Number of Patients	Number of PJP Cases
**1**	Quinn M, et al.	[101]	Memphis, TN, USA	1/2007–8/2014	508 (158 HSCT, 262 ST, 88 L/L) *	0 cases of probable or proven PJP, 4 cases (0.8%) of possible PJP
**2**	Kruizinga MD, et al.	[102]	The Netherlands	5/2011–9/2016	106	1 case of PJP
**3**	Levy ER, et al.	[103]	San Francisco, CA, USA	12/2006–6/2013	111 (all HSCT)	0 cases of PJP
**4**	Solodokin LJ, et al.	[104]	Valhalla, NY, USA	1/2009–7/2014	121	0 cases of PJP
**5**	Curi DA, et al.	[105]	Chicago, IL, USA	1/2007–12/2012	142 (all HSCT)	0 cases of PJP
**6**	Clark A, et al.	[106]	Cincinnati, OH, USA	1/2010–7/2013	333 (all HSCT)	1 case of PJP
**7**	Orgel E, et al.	[107]	Los Angeles, CA, USA	1/2005–12/2010	117	1 case of possible PJP, 1 case of proven PJP
**8**	DeMasi JM, et al.	[108]	Dallas, TX, USA	1/2005–10/2011	137 (all HSCT, 167 transplants **)	0 cases of PJP
**9**	Kim SY, et al.	[109]	USA, Baltimore MA	1/2001–12/2006	232 (106 HSCT)	3 cases of PJP, 2 in HSCT recipients

* HSCT: recipients of HSCT, ST: patients with solid tumors, including central nervous system (CNS) tumors, L/L: patients with leukemia and lymphoma. ** Some patients underwent >1 transplantation procedure.

**Table 3:** Prophylactic regimens for PJP in children with leukemia and HSCT recipients.

Cotrimoxazole *per os* (A-I level of evidence per ECIL-5) * [95]	As trimethoprim (TMP) 5 mg/kg (150 mg/m^2^) and sulfamethoxazole (SMX) 25 mg/kg (750 mg/m^2^), divided once or twice/day for two to three days per week. Maximum daily dose is 320 mg TMP and 1600 mg SMX. ** Duration of prophylaxis is from induction to end of maintenance (ALL), from engraftment to 6 months and while immunosuppression is ongoing (HSCT recipients), and through the duration of chemotherapy in patients with AML and solid tumors.
Pentamidine aerosolized (B-II level of evidence per ECIL-5)	300 mg once a month. To be avoided in children <5 years of age due to concerns of inability to inhale the entire dose. The drug needs to be administered through a Respirgard II jet or similar nebulizer that generates drug particles <4 µm in diameter. Duration of prophylaxis, as per cotrimoxazole.
Pentamidine intravenously (C-II level of evidence per ECIL-5)	4 mg/kg/day, once/day every 28 days. Duration of prophylaxis, as per cotrimoxazole.
Dapsone *per os* (C-II level of evidence per ECIL-5)	2–4 mg/kg/day. Maximum daily dose 100 mg divided twice/day (50 mg bid). Duration of prophylaxis, as per cotrimoxazole.
Atovaquone *per os* (B-II level of evidence per ECIL-5)	30 mg/kg/day for patients aged 1–3 months and 3–12 years. In infants aged 4–24 months, the dose is 45 mg/kg/day. It is administered with a meal as a single daily dose. For adolescents ≥13 years and adults, the dose is 1500 mg once/day. Duration of prophylaxis, as per cotrimoxazole.

* ECIL-5: European Conference on Infections in Leukemia, Version 5, ** Smaller doses, that is, TMP 3 mg/kg (90 mg/m^2^) and SMX 15 mg/kg (450 mg/m^2^) divided twice/day have been successfully used daily or three days per week in the Netherlands.

**Table 4:** Therapeutic regimens for PJP in children with leukemia and HSCT recipients.

Cotrimoxazole (TMP/SMX)	15–20 mg/kg/day trimethoprim (TMP) (maximum TMP dose 960 mg/day) and 75–100 mg/kg/day sulfamethoxazole (SMX) orally or intravenously divided every 6–8 h for 21 days
Clindamycin plus primaquine	Clindamycin 30–40 mg/kg/day orally or intravenously divided every 6–8 h (maximum single dose 600 mg) and primaquine 0.25 mg/kg/day (maximum 15 mg/day) for 21 days
Pentamidine	4 mg/kg/day IV once/day (maximum 300 mg/day) for 21 days
Atovaquone	30 mg/kg/day orally once/day in children <3months and >24 months and 45 mg/kg/day orally divided every 12 h in children 4–24 months for 21 days
